# Joint Measurements of Leukocyte Elastase and Myeloperoxidase Promote Identification of the State of Neutrophils in Diabetic Patients

**DOI:** 10.1089/biores.2020.0012

**Published:** 2020-08-20

**Authors:** Michael Alexandrovski, Soimita Suciu, Jakob Alexandrovski

**Affiliations:** ^1^ Spital Region Oberaargau Langenthal, Langenthal, Switzerland.; ^2^ Department of Physiology, Cluj-Napoca University of Medicine and Pharmacy, Cluj, Romania.; ^3^ Synlab MVZ Ettlingen, Ettlingen, Germany.

**Keywords:** diabetes mellitus, elastase, myeloperoxidase, neutrophils

## Abstract

The clinic of diabetes mellitus (DM) offers a number of hypotheses about the leading role of polymorphonuclear neutrophils (PMNs) in both oxidative stress and diabetic complications. However, the results of numerous studies are extremely controversial. Why is it so? We appreciated the clinical significance of simultaneous measurement data of several PMN parameters, which must complement each other. For this purpose, myeloperoxidase (MPO) and elastase (EL) were jointly analyzed in the blood plasma from 160 type 2 diabetes mellitus patients with high levels of HbA_1c_. A weakly positive correlation (*r* ∼ 0.56) was observed between MPO and EL analytical data, and any correlation between the concentrations of MPO/EL and HbA_1c_ was absent. Medians of 160 measurements of MPO/EL concentrations were ∼103/190 ng/mL, and 95% of all results were in the range below 320/1016 ng/mL, respectively. The share of DM patients whose concentrations of MPO, EL, or either of two parameters exceeded the corresponding reference values was 65%, 80%, and 82.5%, respectively. These findings—a high intensity of neutrophil degranulation process—indicated that some diabetic conditions promote the transfer of PMNs to an “arousal” or “subactivation” state, which is identical or similar to their activation, providing *in vivo* an almost inexhaustible source of extremely “aggressive” MPO and EL. Thus, the conjoint MPO/EL measurements confirm the leading role of PMNs in the development of various complications of diabetes. The paradox is that the diagnostic significance of MPO/EL as independent parameters in diabetic patients is unambiguous for a number of reasons.

## Introduction

In practical diabetology, besides glycated hemoglobin (HbA_1c_), there are very few biomarkers which could reflect an imbalance in carbohydrate metabolism.^1^

For pathologies with the rapid dynamics of inflammatory processes (for example, a postoperative state^2,3^), polymorphonuclear leukocytes (PMNs or neutrophils) are not only participants but also potential witnesses of the actual pathology state. On the other hand, PMNs as biomarkers are very attractive objects of research, largely due to the numerous and relative simple monitoring methods. Consequently, if PMNs are involved in the pathogenesis of any disease, these cells could be tried as biomarkers. Diabetes mellitus (DM) is one such illnesses.^4^

The clinic of DM allowed for the suggestion of a number of hypotheses about the leading role of neutrophils in the development of both oxidative stress and diabetic complications. However these hypotheses are not sufficiently supported by reliable analytical results. Generally the data about the integral evaluation of neutrophils' functional state by DM patients are surprisingly contradictory. It is difficult to choose any functional PMN parameter, which would unambiguously depend on either the level of diabetes compensation or disease *per se*. Moreover even the data about certain PMN protein content in the blood of diabetics are highly ambivalent.

In our studies, we tried to understand the causes of this phenomenon. Using the blood plasma of type 2 diabetes mellitus (T2DM) patients, we performed a parallel analysis of the two most studied PMN enzymes: myeloperoxidase (MPO) and elastase (EL). MPO, for example, can cause a number of pathological processes, such as the formation of reactive oxygen species or hypochlorous acid (HOCl), inducing vascular damage and endothelial dysfunction.^5–8^ EL, for its part, may be responsible for the specific inactivation of C1-inhibitor or antithrombin III: the main regulatory proteins of complement and coagulation systems.^9,10^ By and large, there are many single studies with one or other PMN-derived proteins which indicate their potential implications by diabetic conditions. Therefore, it was hopeful that the conclusions drawn from joint studies of two independent markers could both confirm and complement each other. As a result, we will be able to **(1)** evaluate the significance of MPO and EL as biomarkers by diabetes and **(2)** appreciate the functional state of neutrophils.

## Materials and Methods

We have studied two groups of patients with T2DM in compensated (HbA_1c_ <5.9%) and noncompensated (HbA_1c_ >7.1–14.7%) states. EDTA-plasma from these patients was received from diagnostic laboratory **Synevo** (Cluj-Napoca, Romania) after the necessary routine research. The clinical samples recieved were de-identified and no IRB approval was required for further studies. Thus, we have obtained blood plasma with the following well-known characteristics: the concentrations of glucose and HbA_1c_. Blood plasma was aliquoted in 100 μL plastic microtubes and stored frozen at −25°C until use.

Quantitative determinations of MPO and EL concentrations in blood plasma were carried out using enzyme immunoassay tests (ELISA) provided by the **BioVendor** Company (Brno, Czech). All experiments were performed according to the manufacturer's instructions.

## Results and Discussion

Numerous data concerning the state of “diabetic” PMNs are surprisingly contradictory. We have tried to touch on and understand this problem. It was decided to conduct joint measurements of two different PMN proteins, each of which is capable of characterizing the PMN state by itself. It was hoped that conclusions based on the results of two independent and parallel studies could both confirm and complement each other.

**FIG. 1.**
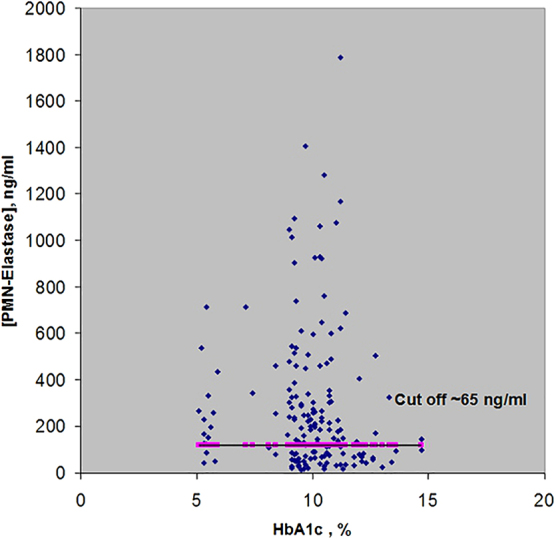
Concentrations of PMN-EL in plasma of 160 patients with good (HbA_1c_ <5.9%) and poorly controlled (HbA_1c_ 7.1–14.7%) T2DM. EL, elastase; HbA_1c_, glycated hemoglobin; PMN, polymorphonuclear leukocyte, neutrophils; T2DM, type 2 diabetes mellitus.

**FIG. 2.**
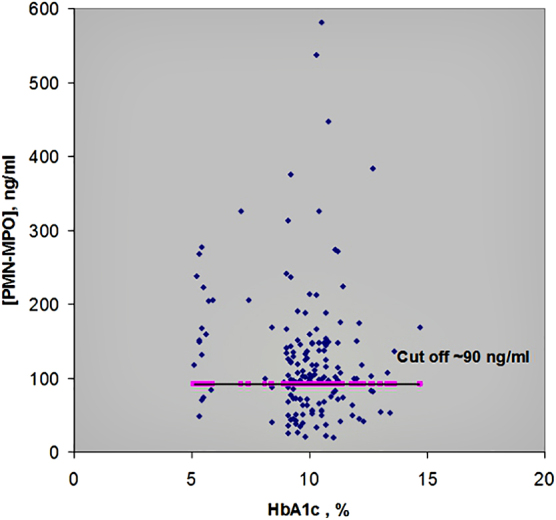
Concentrations of MPO in plasma of 160 patients with good (HbA_1c_ <5.9%) and poorly controlled (HbA_1c_ 7.1–14.7%) T2DM. MPO, myeloperoxidase.

Table 1 shows the concentrations of MPO and EL detected in plasma specimens of 160 patients with T2DM. Sixteen samples were obtained from patients with compensated disease (HbA_1c_ <5.9%, Nr. 1–16) and 144 samples from patients with poorly compensated illness (HbA_1c_ >7.1%; Nr. 17–160). The data in Table 1 are arranged in accordance with the growth of HbA_1c_ concentrations in the samples. The results are also represented graphically in Figures 1 and 2, demonstrating that correlation between the measured concentrations of MPO/EL and the corresponding concentrations of HbA_1c_ was absent. The concentrations of MPO and PMN-EL in the plasma of healthy donors (*n* = 4) varied in the selfsame range from 22 to 65 ng/mL.

**Table 1.** PMN-Elastase and PMN-Myeloperoxidase Concentrations in EDTA-Plasma of Good (*n* = 16) and Poorly (*n* = 144) Controlled T2DM Patients

Nr.	HbA_1C_, %	Elastase, ng/mL	MPO, ng/mL	Nr.	HbA_1C_, %	Elastase, ng/mL	MPO, ng/mL	Nr.	HbA_1C_, %	Elastase, ng/mL	MPO, ng/mL	Nr.	HbA_1C_, %	Elastase, ng/mL	MPO, ng/mL
1	5.1	267	118	41	9.2	238	98	81	10.0	91	97	121	10.7	116	116
2	5.2	538	238	42	9.2	231	122	82	10.0	259	105	122	10.8	492	447
3	5.3	230	152	43	9.2	56	44	83	10.0	303	160	123	10.8	602	97
4	5.3	128	149	44	9.2	1095	121	84	10.0	597	99	124	10.8	308	149
5	5.3	170	268	45	9.3	142	129	85	10.0	276	214	125	10.9	152	75
6	5.3	45	48	46	9.3	330	135	86	10.1	212	118	126	11.0	1078	81
7	5.4	125	168	47	9.3	86	45	87	10.1	262	147	127	11.0	33	20
8	5.4	714	277	48	9.3	739	93	88	10.1	92	57	128	11.1	179	102
9	5.4	89	132	49	9.3	539	97	89	10.1	65	56	129	11.1	226	274
10	5.4	87	71	50	9.3	50	73	90	10.1	199	52	130	11.1	138	83
11	5.5	334	223	51	9.3	460	85	91	10.1	926	148	131	11.2	1786	2058
12	5.5	153	74	52	9.4	64	73	92	10.1	210	103	132	11.2	187	114
13	5.6	196	159	53	9.4	46	51	93	10.2	146	111	133	11.2	36	72
14	5.7	260	205	54	9.4	136	95	94	10.2	132	105	134	11.2	622	272
15	5.8	50	84	55	9.4	28	38	95	10.3	931	118	135	11.3	150	176
16	5.9	437	206	56	9.4	32	43	96	10.3	42	33	136	11.3	20	42
17	7.1	713	326	57	9.5	611	151	97	10.3	185	213	137	11.3	84	108
18	7.4	343	206	58	9.5	14	27	98	10.3	1061	537	138	11.2	114	97
19	8.1	109	99	59	9.5	194	108	99	10.3	463	167	139	11.2	1167	148
20	8.4	255	169	60	9.5	290	191	100	10.4	225	148	140	11.4	36	74
21	8.4	82	41	61	9.5	298	119	101	10.4	923	326	141	11.4	689	224
22	8.4	463	88	62	9.6	20	35	102	10.4	71	66	142	11.8	32	64
23	8.9	165	95	63	9.6	250	97	103	10.4	268	148	143	11.8	71	50
24	9.0	358	141	64	9.6	52	72	104	10.4	237	98	144	11.9	135	100
25	9.0	305	166	65	9.6	132	146	105	10.4	650	138	145	12.0	406	150
26	9.0	1049	242	66	9.6	162	72	106	10.5	29	57	146	12.0	82	100
27	9.0	481	141	67	9.7	449	39	107	10.5	72	55	147	12.1	68	174
28	9.0	240	134	68	9.7	121	64	108	10.5	17	50	148	12.1	51	45
29	9.1	31	26	69	9.7	35	51	109	10.5	1282	98	149	12.2	84	118
30	9.1	1016	313	70	9.7	1405	103	110	10.5	762	582	150	12.3	44	42
31	9.1	283	36	71	9.7	73	110	111	10.6	88	138	151	12.6	58	83
32	9.1	545	88	72	9.8	250	133	112	10.6	43	68	152	12.6	66	103
33	9.1	23	50	73	9.8	340	188	113	10.6	474	102	153	12.7	173	82
34	9.1	327	68	74	9.8	225	102	114	10.6	115	148	154	12.7	505	384
35	9.1	57	126	75	9.8	22	21	115	10.7	216	144	155	13.0	24	54
36	9.1	89	104	76	9.8	509	125	116	10.7	354	154	156	13.3	325	108
37	9.2	515	78	77	9.9	232	136	117	10.7	185	22	157	13.4	49	53
38	9.2	905	376	78	9.9	61	64	118	10.7	303	37	158	13.6	96	136
39	9.2	79	143	79	9.9	187	127	119	10.7	78	125	159	14.7	146	169
40	9.2	387	237	80	9.9	201	72	120	10.7	333	188	160	14.7	100	93

HbA_1c_, glycated hemoglobin; MPO, myeloperoxidase; PMNs, polymorphonuclear leukocytes, neutrophils; T2DM, type 2 diabetes mellitus.

**FIG. 3.**
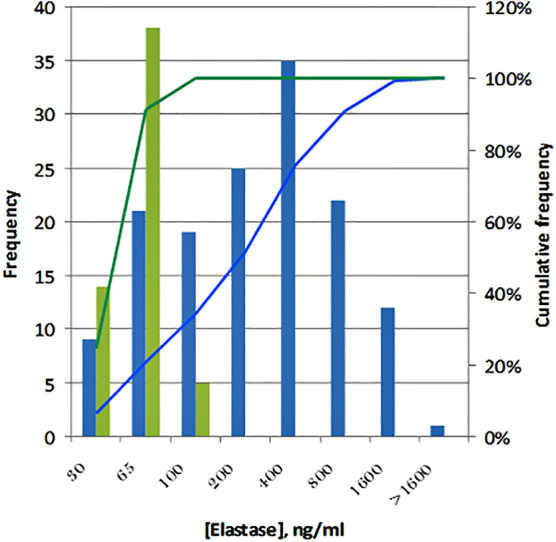
Frequency distribution of PMN-EL concentrations in plasma of healthy (green) donors* and decompensate (blue) T2DM patients**. *Results from BioVendor; **Own research data (Table 1).

ELISA kits and the data of neutrophil-derived EL levels in plasma of healthy people were provided by BioVendor Company: the median concentration of EL was 35 ng/mL and 95% of all measurements were distributed in the concentration range <65 ng/mL. The values of the similar parameters in the plasma of patients with T2DM were ∼190 and 1016 ng/mL, respectively. Based on these data, it was estimated that >80% of patients with diabetes had elevated concentrations of EL in blood. Figure 3 represents the frequencies of EL concentration distribution in the plasma samples of healthy probands and patients with T2DM.

Of course, the elevated levels of EL in blood may not necessarily only be due to the diabetic conditions, but do always indicate the presence of persistent inflammatory processes *in vivo* owing neutrophil activation. To verify this conclusion, MPO measurements were performed in the same clinical samples.

**FIG. 4.**
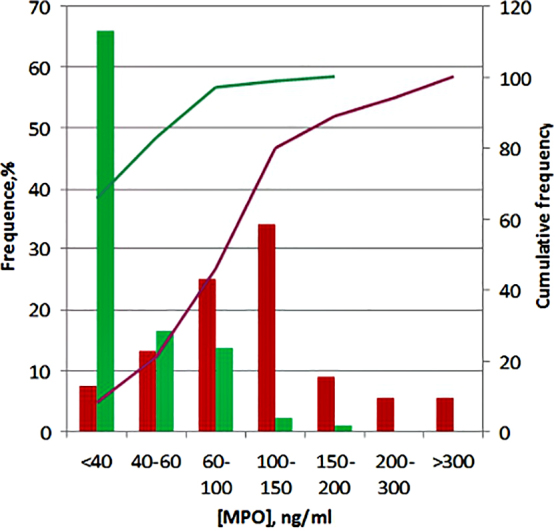
Frequency distribution of MPO concentrations in plasma of healthy (green) donors* and decompensate (red) T2DM patients**. *Results from Ref.^11^; **Own research data (Table 1).

The detected concentrations of MPO in control plasma samples from healthy people were in the same range as in a number of publications.^11–14^ These results confirmed our decision to choose the recommended 95% reference limit of MPO concentrations ∼90.5 ng/mL. This value was calculated after testing 820 plasma samples of healthy people to “provide a scientific basis for the further use of MPO in clinical practice.”^11^ As in the case with EL, MPO concentrations in blood, which exceeded this reference value, could be considered an indicator of the presence of *in vivo* inflammatory processes and infection, or the occurrence of pathologies associated with the activation of neutrophilic leukocytes. The median concentration of MPO in the blood of patients with poorly compensated T2DM was ∼103 ng/mL, and 95% of all measurements were in the concentration range less than ∼320 ng/mL. The quota of diabetic patients with MPO increased levels was ∼65%. To visualize the boundary, which separates healthy and pathological, we compared the frequencies of MPO concentration distribution in the plasma from healthy probands and diabetic patients (Fig. 4).

**FIG. 5.**
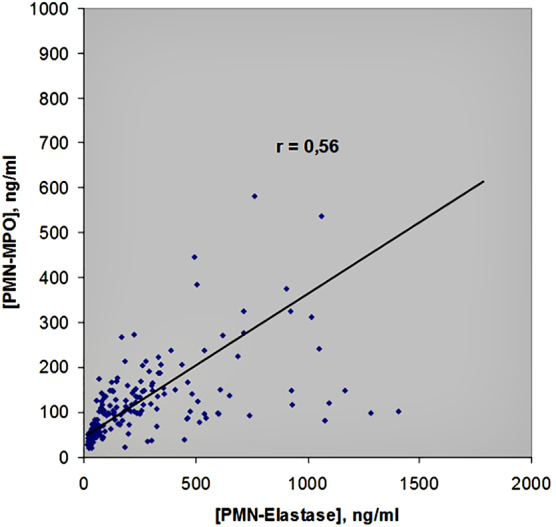
Correlation between MPO and PMN-EL concentrations in plasma of 160 patients with T2DM. Sixteen patients have HbA_1c_ <6.0% and 144 patients characterized with HbA_1c_ from 7.0% to 14.7% (Table 1).

By and large, MPO and EL are usually considered indicators of nonspecific immunity state. As well known, after neutrophil activation, both MPO and EL enzymes leave the azurophilic granules of white blood cells and enter the extracellular space. Therefore, their concentrations in the blood should be approximately equal and correlated with each other. Indeed, many similar studies in healthy people prove this. From these positions, our results were surprising and characterized by the following two remarkable features: first of all, the 95% confidence range of EL concentration distribution in plasma samples from diabetic patients was **more than three times as wide** (!), as for MPO. Second, the correlation coefficient between the steady-state concentrations of MPO and EL was only 0.56 (Fig. 5). The value obtained made it possible to assert the existence of only **moderate positive correlation** between the parameters studied.

Based on these observations, it was suggested that **diabetic condition could somehow either affect the concentration of the studied enzymes or interfere with the ELISA reaction itself**, distorting the analytical results. We tried to identify these processes and evaluate their influence. From this point of view, primarily, the following two unique properties of MPO should be taken into account:

- First of all, MPO is an autoantigen. This MPO feature was confirmed, for example, by the formation of corresponding antineutrophil cytoplasm antibodies (ANCA) in different vasculitis and a number of other rare (compared with T2DM) disorders. Respectively, chronically elevated MPO concentrations by T2DM can also cause sensitization of the body and, as a consequence, the formation of autoantibodies.^15–19^ For example, autoantibodies against MPO were detected in 40% of patients with T2DM.^16^ Generally, the simultaneous presence of both antigen and corresponding autoantibodies in samples can have a disruptive effect on the correctness of ELISA results.^20^
- Another characteristic property of MPO is its ability to adsorb on the walls of blood vessels.^21^ **This MPO feature was reported in** a number of **clinical trials:** after heparin administration, the blood MPO concentrations were greatly increased (sometimes up to two times), while concentrations of EL remained unchanged.^2,22^ In a series of **experiments with cultured human endothelial cells**, it was also shown that diabetes *per se*, or high glucose concentrations, contributed to phenotypic changes of the cell surfaces and caused accelerated sorption processes for both MPO and PMNs.^23^ These findings were also clearly confirmed **by studying microvessels from rats with experimental diabetes**.^7,8^

The process of PMN-EL occurrence in the blood has also its own remarkable features.

First, like MPO, neutrophilic EL under certain conditions can also play the role as an autoantigen.^24^ However, we could not find any reliable information about the presence of EL-specific autoantibodies in DM or about their influence on the results of EL enzyme immunoassay analysis.

From these positions, more attention should be paid to another property of EL. The fact is that once in the extracellular space, EL does not remain intact, and instead builds complexes with some proteins from the so-called “serpin family” (serine protease inhibitors). This interaction really affects *in vivo* both the concentration and enzyme activity. Therefore, taking into account the assumption made, we also evaluated the effect of this phenomenon on EL analyses.

Among the broad family of EL inhibitors, the most active is α1-antitrypsin. Its content in norm (∼1 mg/mL) is at least 10,000 times higher than the baseline level of EL in the blood. Consequently, the concentrations of the complexes formed must be correlated with concentrations of EL released from neutrophils. The BioVendore ELISA-Kit for quantitative analysis of PMN-EL is based on this principle and allows analyzing EL in the plasma samples up to 1000 ng/mL. In this regard, it should be emphasized that, although the clinical material under study was obtained mainly from patients with poorly compensated diabetes, 95% of all results were also located in concentration range <1016 ng/mL. Besides, the measured EL values, as well as the range of their concentration distribution in diabetics, were in good agreement with the results of previous studies.^25^ Therefore, taking into account that PMN-EL does not adsorb on the surface of epithelium cells, we have concluded that the measured data of EL concentrations are reliable and correct.

Unfortunately, a similar proposition cannot be used to evaluate the results of MPO measurements—a widespread research object in many disorders, inclusive diabetes.^4^ The fact is that due to the unique ability to be adsorbed on the walls of blood vessels, the MPO analytical data most likely are not able to fully reflect the true picture of the enzyme content *in vivo*. The degree of sorption will depend on many factors that are difficult to take into account. Therefore, even in the case of a massive influx of MPO into the bloodstream, the measured MPO concentrations in T2DM patients can be underestimated in comparison with real ones. Perhaps for this reason, the values and range of MPO concentrations in patients with diabetes did not deviate too far from reference data. The same results were also represented in many other similar studies.^26–28^ At the same time, we observed increased concentrations of MPO in 65% of patients with DM.

Based on the foregoing, it should be assumed that more valuable information about MPO through diabetes can be obtained by measuring not the enzyme concentration in blood, but its activity in biopsy samples. In this case, the magnitude of the analytical signal will depend not only on the enzyme dissolved but also adsorbed (immobilized) on the walls of the microvasculature. Indeed, in experiments with tissue pieces from rats with experimental diabetes, it was shown that the ratio of specific activities of MPO diabetes/control/g of tissue reached >300%.^29^ Naturally, the practical use of this approach is unacceptable. However, these data allow us to estimate approximately the degree of MPO sorption and notice its significance in diabetes. Of course, this topic requires special research. However, it can already be argued that MPO adhesion *in vivo* should proceed more intensively in the capillary network due to the significant increase of the inner blood vessel surface (and, consequently, an increase of sorption area). It is highly likely that this mechanism is dominant in the pathology of diabetic angiopathy, especially considering the significantly extended lifetime of immobilized MPO *in vivo* (see the following discussion).

Thus, we were able to identify several potential causes, which could significantly affect the ratio of MPO/EL concentrations *in vivo*. Among them, the main cause is solely the polycationic nature of MPO, which ensures its strong interactions with negatively charged surfaces of endothelial cells.^21^

From this point of view, the use of EL as a biomarker seems to be a more reliable and informative parameter. However, it is well known that an increase of PMN-EL in the blood could be associated not only with diabetes but also with a large number of other pathologies. Therefore, in the absence of an adequate reference parameter, which will be specific especially for diabetes, it is unlikely to use only solo EL measurements as a nonspecific biomarker for mass use.^25^

In fact, this statement applies not only to EL but also to the neutrophils themselves or any other PMN proteins when trying to use them as diabetic biomarkers. That is why many researchers tried through diabetics to find the relationship between the different characteristics of PMN and HbA_1c_ levels. However, some discovered relationships between HbA_1c_ and, for example, MPO activity,^30,31^ amounts of neutrophil leukotriene B4,^32^ or activity of PMN-alkaline phosphatase^33^ strangely did not coincide with the results of similar^26,34^ or present studies.

Apparently, the absence of such correlation between the concentrations of MPO/EL and HbA_1c_ in patients with T2DM can be explained if we take into account the time hierarchy of the processes under consideration and compare the life cycle durations of their participants (Table 2). As follows from the data presented in Table 2, during the lifetime of one generation of HbA_1c_ *in vivo*, at least several tens of PMN generations will be replaced. In other words, substitution of MPO or EL concentrations in blood will proceed much faster than the changes of HbA_1c_ concentrations. Consequently, the content of HbA_1c_ *in vivo* in the framework of supposed correlations must be considered not as a variable, but as a constant value. This means that, even theoretically, **there is no and cannot be any relationship** between HbA_1c_ and MPO/EL activity/concentrations.

**Table 2.** Half-Life of PMNs and Certain Proteins Involved in Diabetes Pathology

Name	Half-life, τ½	References
HbA_1c_	∼28.7 days (689 h)	^35^
Fructosamine	∼16.5 days (396 h)	^35^
PMNs in blood	∼6–7 h	^36^
PMN elastase in complex with α2-macroglobulin	∼12 min (0.2 h)	^37^
PMN elastase in complex with α1-proteinase inhibitor	∼60 min (1 h)	
MPO in blood	∼4 min	^7,8^
MPO immobilized on vascular wall	∼4320 min (3 days)	

For these reasons, the results of some recent studies, which have also indicated a positive correlation between HbA_1c_ levels and concentrations of certain proinflammatory proteins (with a clearly shorter life span than hemoglobin), such as angiopoietin-like protein-6^38^ or interleukin-6^39^ should be carefully reconsidered.

## Conclusions

The joint measurements of MPO and EL can independently characterize the same one state of neutrophils from different “points of view.” This allowed us to compare analytical meaningfulness of both the tests used and to conclude about the PMN status in patients with T2DM. It was shown that (1) the quota of patients with elevated levels of MPO and EL were 65% and 80%, respectively; (2) between measured concentrations of MPO and EL was observed only a weak correlation, and (3) the 95% range of MPO distribution among diabetic patients was only slightly different from the control and was almost three times less wide as for the EL.

To evaluate the results obtained, it was necessary to take into account not only the physicochemical properties of MPO but also mechanism of its biological activity. Namely the nature of MPO determined its “dualistic role” *in vivo*—on the one hand, the ability to simultaneously protect the organism from various types of bacterial infection and, on the other hand, to attack the capillary network of blood circulation under certain conditions. This phenomenon is based on the same one mechanism of MPO action: at first stage, the polycationic enzyme globules “adhere” to the negatively charged surfaces of both different pathogens or endothelial cells. This process can lead to the depletion of MPO in the bloodstream even during powerful enzyme inflow from activated neutrophils. That is why MPO concentrations in diabetic patients may not differ significantly from normal values and the quota of DM patients with elevated MPO levels was depressed compared with EL test.

At the same time, the joint MPO and EL measurements unequivocally indicated the high intensity of PMNs degranulation processes in the blood of diabetic patients. That means that diabetes promotes the transition of neutrophils into a state of “subactivation,” which is identical or similar to the state of activated PMNs. Therefore, it can be argued that MPO and EL really take an active part in numerous pathological processes in diabetes. The paradox is that the diagnostic significance of MPO (especially) or EL as independent solo “diabetic” biomarkers may be in some cases not only uninformative but even erroneous.

## Abbreviations Used

ANCA: anti-neutrophil cytoplasmic antibody
DM: diabetes mellitus
EL: elastase
ELISA: enzyme-linked immunosorbent assay
HbA_1c_: glycated hemoglobin/glycosylated hemoglobin
HOCl: hypochlorous acid
MPO: myeloperoxidase
PMNs: polymorphonuclear leukocytes, neutrophils
T2DM: type 2 diabetes mellitus
